# Structure-guided modification of a lead cholinesterase-targeting oxime to improve peripheral site binding

**DOI:** 10.2478/aiht-2026-77-4078

**Published:** 2026-03-30

**Authors:** Nikola Maraković, Nicolas Probst, Tena Čadež, Antonio Zandona

**Affiliations:** Institute for Medical Research and Occupational Health, Division of Toxicology, Zagreb, Croatia; University of Rouen Normandy, National Institute of Applied Sciences, COBRA Laboratory, Rouen, France

**Keywords:** 2-hydroxyiminoacetamides, acetylcholinesterase, butyrylcholinesterase, cycloaddition, tetrahydroisoquinoline, 2-hidroksiiminoacetamidi, acetilkolinesteraza, butirilkolinesteraza, cikloadicija, tetrahidroizokinolin

## Abstract

Based on our previous study on *N‑*substituted 2-hydroxyiminoacetamides as cholinesterase-targeting ligands, which highlighted 2-hydroxyimino-*N*-(3-(4-((2-methyl-*1H*-imidazol-1-yl)methyl)-*1H*-1,2,3-triazol-1-yl)-1-phenylpropyl)acetamide as lead compound because of its inhibition potency, we extended this group of compounds by introducing a new, bulkier peripheral anionic site (PAS)-binding substituent – 6,7-dimethoxy-1,2,3,4-tetrahydroisoquinoline to achieve an orientation of its three modular elements that would comply with our initial design: the benzyl unit to occupy the choline binding region, the bulky substituent to stabilise binding at the peripheral site, and the 2-hydroxyiminoacetamide moiety to bind at the catalytic serine residue. The obtained *N*-(3-(4-((6,7-dimethoxy-3,4-dihydroisoquinolin-2(*1H*)-yl)methyl)-*1H*-1,2,3-triazol-1-yl)-1-phenylpropyl)-2-(hydroxyimino)acetamide inhibited both human acetylcholinesterase (AChE) and butyrylcholinesterase (BChE) non-competitively in a low micromolar range. Molecular docking studies predicted high stability of the 6,7-dimethoxy-1,2,3,4-tetrahydroisoquinoline group at PAS and of the 2-hydroxyiminoacetamide moiety and the benzyl group inside the catalytic site of BChE, while the closest orientation of the three modular elements to our initial design was predicted for AChE, though different orientations were also predicted. Cytotoxicity was observed in the HepG2 and HEK293 cell lines, but only at concentrations exceeding those used in the reversible inhibition assay. Our study confirms *N*-substituted 2-hydroxiiminoacetamides with three structural elements as low micromolar cholinesterase ligands, successfully extending the scope of PAS substituent to 6,7-dimethoxy-1,2,3,4-tetrahydroisoquinoline. Beyond cholinesterase inhibition, our findings contribute to better understanding of the cytotoxic properties of constituting structural elements of the tested *N*-(3-(4-((6,7-dimethoxy-3,4-dihydroisoquinolin-2(*1H*)-yl)methyl)-*1H*-1,2,3-triazol-1-yl)-1-phenylpropyl)-2-(hydroxyimino)acetamide.

## Abstract (hr)

Ovim radom proširujemo istraživanje *N*-supstituiranih 2-hidroksiiminoacetamida kao liganada usmjerenih na kolinesteraze. Polazeći od prethodnog istraživanja u kojem je 2-hidroksiimino-*N*-(3-(4-((2-metil-*1H*-imidazol-1-il)metil)-*1H*-1,2,3-triazol-1-il)-1-fenilpropil)acetamid istaknut kao vodeći spoj zbog svoje inhibitorske učinkovitosti, proširili smo ovu skupinu spojeva uvođenjem novog, glomaznijeg supstituenta koji se veže u periferno alosteričko mjesto – 6,7-dimetoksi-1,2,3,4-tetrahidroizokinolina kako bi se postigla orijentacija triju modularnih elemenata u skladu s početnim dizajnom: benzilne jedinice koja zauzima kolinski vezni džep, glomaznog supstituenta koji stabilizira vezanje u perifernom mjestu te 2-hidroksiiminoacetamidne skupine, usmjerene prema katalitičkom serinu. Ispitivanja inhibicije pokazala su da *N*-(3-(4-((6,7-dimetoksi-3,4-dihidroizokinolin-2(*1H*)-il)metil)-*1H*-1,2,3-triazol-1-il)-1-fenilpropil)-2-(hidroksiimino)acetamid inhibira ljudske AChE i BChE u niskom mikromolarnom rasponu, pri čemu iskazuje nekompetitivni način inhibicije. Molekulskim pristajanjem predviđeno je da je skupina 6,7-dimetoksi-1,2,3,4-tetrahidroizokinolina dobro stabilizirana u perifernom mjestu, a 2-hidroksiiminoacetamidna skupina i benzilna skupina smještene unutar katalitičkog mjesta u slučaju BChE, dok se u slučaju AChE predviđa najsličnija orijentacija triju modularnih elemenata našem početnom dizajnu, premda su predviđene orijentacije raznovrsnije. Mjerenja citotoksičnosti pokazala su da je pogođena jedino vijabilnost humanih staničnih linija HepG2 i HEK293, i to pri koncentracijama iznad raspona koji je korišten u ispitivanjima reverzibilne inhibicije. Naše istraživanje potvrđuje da su *N*-supstituirani 2-hidroksiiminoacetamidi s trima strukturnim elementima ligandi kolinesteraze u niskom mikromolarnom području, pri čemu je uspješno proširen raspon supstituenata za periferno mjesto na 6,7-dimetoksi-1,2,3,4-tetrahidroizokinolin. Osim inhibicije kolinesteraze, naši nalazi pridonose boljem razumijevanju citotoksičnih svojstava strukturnih elemenata koji čine ispitivani *N*-(3-(4-((6,7-dimetoksi-3,4-dihidroizokinolin-2(1*H*)-il)metil)-1*H*-1,2,3-triazol-1-il)-1-fenilpropil)-2-(hidroksiimino)acetamid.

Acetylcholinesterase (AChE; EC 3.1.1.7) is essential for cholinergic neurotransmission because it rapidly hydrolyses acetylcholine at synapses in the central and peripheral nervous systems. Disturbances in this pathway can have both toxic and therapeutic outcomes. In Alzheimer’s disease (AD), deficits in cortical and hippocampal cholinergic tone contribute to memory loss and learning impairment, which has prompted the search for reversible AChE inhibitors that could elevate synaptic acetylcholine and relieve AD symptoms, at least temporarily (1,2,3,4). For now, only donepezil, galantamine, and rivastigmine have been approved for symptomatic treatment by blocking the enzyme’s active site to slow down acetylcholine turnover.

Like AChE, butyrylcholinesterase (BChE; EC 3.1.1.8) has increasingly been recognised as a co-regulator of cholinergic signalling in the brain, although its precise physiological role remains to be seen. Because of its ability to hydrolyse acetylcholine, it too may play a role in the pathogenesis and progression of AD, but unlike AChE, BChE activity is preserved or even elevated in AD patient’s cortex, which implies that BChE gradually takes over acetylcholine hydrolysis as the disease progresses (5, 6). This shift informs therapeutic strategies that consider both enzymes in modulating cholinergic tone.

A second arena where cholinesterases are central is organophosphorus (OP) poisoning, either by OP pesticides or warfare nerve agents, which covalently phosphylate the catalytic serine of AChE, produce an inactive conjugate, and precipitate cholinergic crisis. Standard medical countermeasures combine antimuscarinic and anticonvulsant drugs with a nucleophilic oxime reactivator designed to cleave the phosphorus-oxygen bond to serine and restore enzymatic activity (7,8,9). Yet the efficiency of oximes is limited by the steric and electronic features of the AChE-OP conjugate, and current oximes differ markedly in activity across OP structures. In addition, the quaternary ammonium of classical pyridinium oximes limits their penetration into the central nervous system (CNS), preventing effective reactivation of brain AChE (10).

Challenged by these limitations, we explored structure-based strategies for ligands that engage the enzyme more productively and, when uncharged, have the potential to act in the CNS. Building on overlays of AChE-ligand crystal structures, we delineated the geometry of a narrow 20 Å-deep catalytic gorge and mapped three regions with a potential for interaction: the choline binding region within the catalytic anionic subsite (CAS) at the base, the peripheral anionic subsite (PAS) near the gorge entrance, and the space between the catalytic triad and oxyanion hole that becomes occupied by the phosphyl group after enzyme inhibition by OP. Guided by these features, we selected modular elements to span the gorge and engage with each of the three mapped regions. The benzyl unit was to engage the choline binding region, the peripheral substituent to stabilise binding at the PAS, and the 2-hydroxyiminoacetamide moiety to act as the reactivating group in case of OP poisoning (11, 12).

Among the synthesised *N*-substituted 2-hydroxyimino acetamides, 2-hydroxyimino-*N*-(3-(4-((2-methyl-*1H*-imidazol-1-yl) methyl)-*1H*-1,2,3-triazol-1-yl)-1-phenylpropyl)acetamide (**1**; Figure 1A) emerged as a lead. Despite its modest reactivation efficiency, **1** turned out to be a potent enantioselective inhibitor of human BChE, with the dissociation constant (*K*_I_) in low micromolar range and, to a lesser extent, of human AChE (13). Crystallographic analysis of human BChE complexed with **1** (Figure 1B) confirmed the anticipated triple-binding mode. The methylimidazole ring stacked with Trp82, the benzyl group engaged a hydrophobic region including Asp70, the triazole sit between Phe329 and Tyr332, and the oxime oriented towards the catalytic serine.

**Figure 1:**
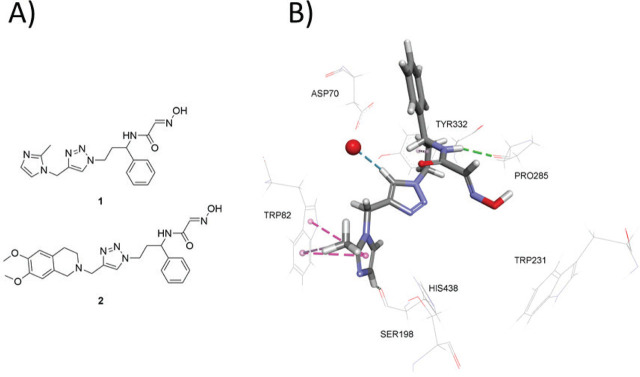
A) *N*-substituted 2-hydroxyiminoacetamides **1** and **2**; B) crystallographic structure of oxime *(R)*-**1** bound to human BChE (PDB: 6T9P). Non-covalent interactions are shown: hydrogen bonds (green dashed lines) and π interactions (magenta dashed lines). **1** – 2-hydroxyimino-*N*-(3-(4-((2-methyl-1*H*-imidazol-1-yl)methyl)-1*H*-1,2,3-triazol-1-yl)-1-phenylpropyl)acetamide; **2** – *N*-(3-(4-((6,7-dimethoxy-3,4-dihydroisoquinolin-2(1*H*)-yl)methyl)-1*H*-1,2,3-triazol-1-yl)-1-phenylpropyl)-2-(hydroxyimino)acetamide

To further improve the binding of **1** and contribute to the development of much needed selective BChE inhibitor for symptomatic AD treatment, we sought to introduce a more elaborate PAS-binding substituent into the core *N*-(3-azido-1-phenylpropyl)-2-hydroxyiminoacetamide structure. For that purpose, we turned our attention to 6,7-dimethoxy-1,2,3,4-tetrahydroisoquinoline. Namely, tetrahydroisoquinoline (THIQ)-based aldoximes are known multifunctional ligands which can be tailored for brain delivery and robust enzyme engagement (14) but also possess cytotoxic properties via mitochondria-mediated apoptosis (15). These findings prompted us to look at THIQ aldoximes not only as candidates for AChE and BChE ligands but also as leads for studying oncological applications in which targeted proapoptotic activity is desirable.

The aim of this study was to see if the newly designed and synthesised *N*-(3-(4-((6,7-dimethoxy-3,4-dihydroisoquinolin-2*(1H)-*yl)methyl)-*1H*-1,2,3-triazol-1-yl)-1-phenylpropyl)-2-(hydroxyimino) acetamide (**2**; Figure 1A; hereinafter referred to as the *test compound*), which combines the traits of the previously identified lead compound and the ubiquity of THIQ, would exhibit even higher inhibition potency against AChE and BChE. Additionally, we hoped that the collected information on its cytotoxic properties would shed more light on the cellular pharmacology of THIQ aldoximes.

## MATERIALS AND METHODS

### Chemicals and equipment

All reactions were carried out under an inert argon atmosphere, unless stated otherwise. Tetrahydrofuran (CAS No. 109-99-9) was distilled over lithium aluminium hydride. All other solvents were used in commercial formulation without further purification. All reagents were obtained from Sigma-Aldrich (St. Louis, MO, USA). Thin layer chromatography (TLC) was run on aluminium-backed silica plates (60 F254, Merck, Darmstadt, Germany) and visualised using UV light (254 nm) or phosphomolybdic acid reagent. Column chromatography was performed on silica gel (Silicagel 60, 70–230 mesh, Merck) or flash silica gel (Silicagel 60, 230–400 mesh, Merck).

^1^H and ^13^C nuclear magnetic resonance (NMR) spectra were recorded on a Bruker Avance 600 spectrometer (Bruker, Millerica, MA, USA) in CDCl_3_. Chemical shifts (δ) are given in ppm and are referenced to tetramethylsilane or the internal protic solvent.

High-performance liquid chromatography (HPLC) was run on a Dionex UltiMate HPLC system (Thermo Fisher Scientific, Waltham, MA, USA) equipped with a diode-array detector (DAD), controlled by the Chromeleon version 7.2.4.8179 chromatography data system (Thermo Fisher Scientific). Detection was set at 254 nm with a 2 nm bandwidth on the UV_VIS_1 channel. Upon 5 µL sample solution injections into the Syncronis aQ column (100 mm length × 3 mm i.d., 3 µm particles), the run time was 41.0 min at a flow rate of 0.50 mL/min. Elution employed a gradient from 100 % solvent A (50 mmol/L ammonium acetate buffer) to 100 % solvent C (acetonitrile). Purity was evaluated by peak-area percentage at 254 nm without correction factors (sum of areas reported as 100 %).

**Figure 2:**
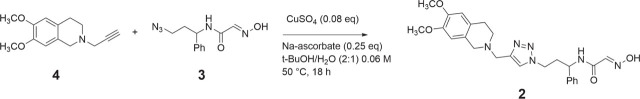
Synthesis of the test compound *N*-(3-(4-((6,7-dimethoxy-3,4-dihydroisoquinolin-2(1*H*)-yl)methyl)-1*H*-1,2,3-triazol-1-yl)-1-phenylpropyl)-2-(hydroxyimino)acetamide. **2** – *N*-(3-(4-((6,7-dimethoxy-3,4-dihydroisoquinolin-2(1*H*)-yl)methyl)-1*H*-1,2,3-triazol-1-yl)-1-phenylpropyl)-2-(hydroxyimino) acetamide; **3** – *N*-(3-azido-1-phenylpropyl)-2-hydroxyiminoacetamide; **4** – 6,7-dimethoxy-2-(prop-2-yn-1-yl)-1,2,3,4-tetrahydroisoquinoline

*N*-(3-azido-1-phenylpropyl)-2-hydroxyiminoacetamide (**3**; Figure 2) was prepared as reported previously (11, 12). It served as the basis for the test compound in this study, *N*-(3-(4-((6,7-dimethoxy-3,4-dihydroisoquinolin-2(*1H*)-yl)methyl)-*1H*-1,2,3-triazol-1-yl)-1-phenylpropyl)-2-(hydroxyimino)acetamide (**2**), prepared by adding 0.1 mol/L sodium ascorbate (0.50 mL, 0.05 mmol) and 0.1 mol/L CuSO_4_ (0.16 mL, 0.016 mmol) to a solution of 6,7-dimethoxy-2-(prop-2-yn-1-yl)-1,2,3,4-tetrahydroisoquinoline (**4**; Figure 2) (47 mg, 0.20 mmol) and *N*-(3-azido-1-phenylpropyl)-2-hydroxyiminoacetamide (50 mg, 0.20 mmol) in *t*-BuOH/H_2_O (2:1, 3 mL) (Figure 2). The reaction mixture was warmed to 50 °C overnight, cooled to room temperature, and the solvent removed by rotary evaporation. The crude reaction mixture was added 5 mL of 2 mol/L NH_4_OH and extracted with EtOAc (3×25 mL). The organic layer was dried over MgSO_4_ and evaporated to yield the corresponding 1,4-triazole oxime, i.e. the test compound (yield: 56 %), which was then purified in a chromatograph column (0–10 % CH_3_OH in CH_2_Cl_2_ over 30 min on a 12 g high-performance silica cartridge, 20 mL/min) to HPLC purity above 95 %.

### *In vitro* enzyme activity assays

To determine enzyme activity, we used acetylthiocholine iodide (ATCh; Sigma-Aldrich) as substrate dissolved in distilled water and 5,5-dithiobis-2-nitrobenzoic acid (DTNB) (Sigma-Aldrich) dissolved in 0.1 mol/L sodium phosphate buffer (pH 7.4) as reagent following the procedure described by Ellman et al. (16). All chemicals were of analytical purity.

The stock solution of the test compound (200 mmol/L) was prepared by dissolving it in dimethyl sulphoxide (DMSO; Kemika, Zagreb, Croatia) and storing at 4 °C. Further dilutions were made in sodium phosphate buffer prior to experiment. Sources of AChE and BChE were native human erythrocytes and native human plasma, respectively. Enzyme activity was measured spectrophotometrically with the Ellman assay with DTNB (0.3 mmol/L) and substrate ATCh (0.1–0.3 mmol/L) (16, 17) at 25 °C using the Tecan Infinite M200PRO plate reader (Tecan Austria GmbH, Grödig, Austria). Reversible inhibition was measured as described in our previous study (12).

### Molecular docking

Molecular docking was used to predict the orientation and noncovalent interactions of the test compound within the active site of AChE and BChE. The crystal structures used as templates for model human AChE and BChE receptors were the Protein Data Bank (PDB) 4EY4 and 1P0I, respectively (18, 19). Ligand structures were sketched in ChemBio3D Ultra 21.0 (PerkinElmer, Inc., Waltham, MA, USA) and prepared for docking with the Prepare Ligands protocol in BIOVIA Discovery Studio Client version 21 (Dassault Systèmes, Vélizy-Villacoublay, France), which takes into account possible protonation states, stereoisomers, and tautomers at pH 7.4. The ligands were then energy-minimised with the CHARMm force field and the Smart Minimizer algorithm via the Minimize Ligands protocol implemented in BIOVIA Discovery Studio. Before docking, the enzyme receptors were prepared by defining the ligand-binding site within the crystal structures. The binding site was defined as a sphere surrounding the residues bordering the active site gorge (r=13.5 Å for AChE and r=14.0 Å for BChE). Conserved water molecules retained for docking were identified by superimposing water networks from crystal structures of various AChE and BChE complexes available in PDB and then by transferring the conserved waters into the AChE and BChE crystal structures used as receptors. Docking into the receptor active sites was carried out in BIOVIA Discovery Studio using the Flexible Docking protocol. For flexible residues delimiting the active-site gorge we selected those most frequently forming noncovalent interactions with ligands: Tyr72, Trp86, Tyr124, Tyr133, Ser203, Trp236, Trp286, Phe295, Phe297, Glu334, Tyr337, Phe338, Tyr341, His447, and Tyr449 for AChE and Asn68, Asp70, Trp82, Gln119, Tyr128, Glu198, Ser198, Pro285, Leu286, Ser287, Trp231, Glu325, Phe329, Phe398, and His438 for BChE. The cutoff angle for determining whether two side-chain χ1 torsion angles (defined over the N–Cα–Cβ–Cγ atoms) were identical was set to 25 °C. Only conformations in which this torsion angle differed by more than the cutoff were considered distinct. Ligand conformations were generated using the BEST algorithm to ensure maximal coverage of the ligand conformational space. Conformations of individual stereoisomers were generated within a relative energy threshold of 20 kcal/mol. During LibDock-based docking, an RMSD tolerance of 0.25 Å was used to accept or reject a given hotspot match between ligand and receptor conformers. Other LibDock parameters were set to the predefined high-quality settings. The retained ligand orientations were then refined by molecular dynamics, using a simulated annealing scheme in which the temperature was raised to 700 K (426.85 °C; 2000 cycles) and subsequently lowered to 310 K (36.85 °C; 5000 cycles). Each refined ligand pose in the rigid receptor was minimised one last time, after which the interaction energy and CHARMm energy (the sum of the interaction energy and ligand strain) were calculated for each. Representative poses for each enzyme-ligand complex were selected by identifying those with the highest consensus across scoring functions that estimate enzyme-ligand binding affinity.

### Cytotoxicity assessment

Human Caucasian hepatocyte carcinoma HepG2 (ECACC 85011430), human embryo kidney HEK293 (ECACC 85120602), and human neuroblastoma SH-SY5Y (ECACC 94030304) cell lines were obtained from the European Collection of Authenticated Cell Cultures (ECACC) through Sigma-Aldrich (Steinheim, Germany). HepG2 and HEK293 cells were grown in Eagle’s Minimum Essential Medium (EMEM) and SH-SY5Y in Dulbecco’s Modified Essential Medium with F12 nutrient mixture (DMEM-F12) as described previously (20) and cultivated and maintained according to the standard protocol (21). All media and supplements were purchased from Sigma-Aldrich, Steinheim, Germany.

The cytotoxic profile of the test compound was determined by measuring succinate dehydrogenase mitochondrial activity in exposed cells using the commercial CellTiter 96^®^ AQueous One Solution Cell Proliferation Assay (Promega, Madison, WI, USA) based on 3-(4,5-dimethylthiazol-2-yl)-5-(3-carboxymethoxyphenyl)-2-(4-sulfophenyl)-2h-tetrazolium (MTS) colourimetry as described elsewhere (23). Cells were exposed to the test compound in the concentration range of 25–800 µmol/L for 24 h. DMSO (20 %) was used as positive control to validate the method. After the 24-hour incubation at 37 °C in a 5 % CO_2_ atmosphere, cells were washed with a phosphate-buffered saline (PBS) and added 100 µL of EMEM or DMEM and 20 µL of MTS. After incubation of up to 1 h, the absorbance was read at 492 nm on an Infinite M200PRO plate reader (Tecan Austria GmbH). Data are presented as percentages of the inhibited cells to untreated control cells, i.e. as cytotoxicity percentages. IC_50_ values (compound concentrations that kill 50 % of cells) were determined by a nonlinear-fit equation predefined in Prism 9 software (GraphPad Software, San Diego, CA, USA).

## RESULTS

### Enzyme inhibition with the test compound 2

Reversible inhibition measurements revealed non-competitive test compound inhibition of both human AChE and BChE in the micromolar range. With the dissociation constant (*K*_I_) of 18 µmol/L, BChE had higher affinity for the test compound than AChE (49 µmol/L) (Table 1). Compared to the *K*_I_ of the previously obtained lead compound (1) (33 µmol/L for AChE, 4 µmol/L for BChE), the newly synthesised test compound is slightly less potent AChE inhibitor, while the inhibition potency is even lower for BChE. These results indicate that the implemented structural modification of the previous lead compound (1) did not enhance its inhibitory potency. Nevertheless, the test compound (2) retained *K*_I_ in the micromolar range for both enzymes.

**Table 1:** Reversible inhibition of human red blood cell acetylcholinesterase (AChE) and human plasma butyrylcholinesterase (BChE) by the test compound *N*-(3-(4-((6,7-dimethoxy-3,4-dihydroisoquinolin-2(1*H*)-yl)methyl)-1*H*-1,2,3-triazol-1-yl)-1-phenylpropyl)-2-(hydroxyimino)acetamide

**Enzyme**	**Acetylthiocholine (mmol/L)**	**Concentration range (µmol/L)**	***K*_I_ (µmol/L)**
AChE	0.1–1.0	25–75	49±4
BChE	0.1–1.0	5–30	18±1

*K*_I_ – dissociation constant

### Molecular docking results

To predict the binding mode of the test compound inside the AChE and BChE active site, we docked it into the crystal structure of human AChE and BChE using the Flexible Docking protocol, which generates different enzyme conformations with regards to predefined flexible residues. Interestingly, all top scored poses chosen to illustrate different binding modes included the (*R*)-enantiomer of the test compound. This is in line with the low enantioselectivity of the (*R*)-enantiomer in case of both AChE and BChE exhibited by our previous lead compound (1).

**Figure 3:**
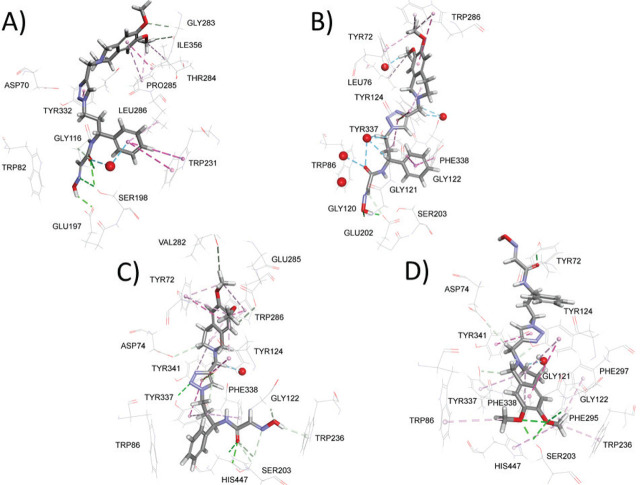
Model complexes of the test compound *N*-(3-(4-((6,7-dimethoxy-3,4-dihydroisoquinolin-2(1*H*)-yl)methyl)-1*H*-1,2,3-triazol-1-yl)-1-phenylpropyl)-2-(hydroxyimino)acetamide and human BChE (A) or human AChE (B–D). Dashed lines represent different types of non-bonding interactions (magenta – π-alkyl, π-π interaction; green – conventional hydrogen bond; light green – carbon hydrogen bond; blue – water hydrogen bond). Red spheres show only those water molecules (hydrogens hidden) predicted to interact with ligands

Figure 3A illustrates the predicted binding mode and non-bonding interactions of the test compound (2) inside the BChE active site. Compared to the crystallographic structure of oxime (*R*)-**1** bound to human BChE (Figure 1B), the test compound adopts a markedly different orientation with both the benzyl group and the 2-hydroxyiminoacetamide moiety occupying the catalytic site, as well as with the newly introduced 6,7-dimethoxy-1,2,3,4-tetrahydroisoquinoline group located at the gorge entrance. The 6,7-dimethoxy-1,2,3,4-tetrahydroisoquinoline group is well-stabilised at PAS via multiple non-bonding interactions, including carbon hydrogen bonds and π interactions with residues Gly283, Ile356, Thr284, and Pro285. Moreover, this orientation promotes interaction between 2-hydroxyiminoacetamide moiety and Ser198 via conventional hydrogen bond, which further stabilises the 2-hydroxyiminoacetamide moiety through interaction with the Gly116 of the oxyanion hole. The only disagreement with our initial design concerns the predicted orientation of the benzyl group inside the catalytic site; instead of occupying the choline binding region near Trp82, it is predicted to orient towards Trp231, with which it interacts via π interactions accompanied by interactions with another acyl pocket residue, Leu286. However, the interaction with Trp231 has been recognised as a common motif in selective BChE inhibitors (22,23,24,25,26).

The molecular docking results regarding the binding of the test compound inside the AChE active site are more diverse. Namely, among the top scored poses, three different orientations stand out (Figure 3B–D). The predicted orientation illustrated by Figure 3B resembles the predicted orientation inside the BChE active site, as the benzyl group is directed towards the acyl pocket, while 2-hydroxyiminoacetamide moiety is directed towards the bottom of the catalytic site but still too distant from Ser203 to engage in interaction. The second distinctive orientation (Figure 3C) retains localisation of the 6,7-dimethoxy-1,2,3,4-tetrahydroisoquinoline group at the gorge entrance but places the benzyl group and 2-hydroxyiminoacetamide moiety inside the catalytic side. The benzyl group is predicted to occupy the choline binding region near Trp82, while the 2-hydroxyiminoacetamide moiety is directed towards the acyl pocket. It therefore engages in hydrogen bonds with Ser203 and His447 from the catalytic triad. This orientation is the closest to our initial design. Finally, the third predicted orientation is characterised by the PAS-binding 6,7-dimethoxy-1,2,3,4-tetrahydroisoquinoline group occupying the catalytic site, while the benzyl group and 2-hydroxyiminoacetamide moiety are directed towards the gorge entrance, stabilised by multiple non-bonding interactions with most of the residues outlining the active site gorge, including Ser203 and His447 from the catalytic triad, but missing the interaction with Trp286, which is a distinct PAS residue (Figure 3D). Altogether, the diversification of the predicted orientations inside the active AChE site implies instability and less-than-ideal complementarity between the test compound and the AChE gorge site.

### Cytotoxicity findings

The obtained IC_50_ values of ≥800 µmol/L for SH-SY5Y, 385±8 µmol/L for HepG2, and 321±22 µmol/L, for HEK293 cell lines indicate that the test compound can only affect the viability of human HepG2 and HEK293 cells in the studied concentration range, but not in the concentration range used for reversible inhibition.

## DISCUSSION

While molecular docking predicts partial agreement with our initial design – benzyl group and 2-hydroxyiminoacetamide moiety occupying catalytic site and 6,7-dimethoxy-1,2,3,4-tetrahydroisoquinoline group located at the gorge entrance – for both ChEs (ideally, this should be confirmed by a crystallographic study), the reversible inhibition measurements show only moderate inhibition of AChE and lower inhibition of BChE than that achieved with our previous lead compound (**1**). One can argue that, in spite of the snug fit between the test compound and BChE active site gorge, it is unclear to which extent docking represents the real situation, i.e., how frequently the test compound enters the BChE active site gorge, given the increased volume with the 6,7-dimethoxy-1,2,3,4-tetrahydroisoquinoline group compared to the methylimidazole ring of the previous compound. However, greater volume would imply even lower AChE inhibition than the one we measured, given that the AChE active site gorge is 200 Å^3^ smaller. One should bear in mind that our initial structure-guided design of triple-binding oxime reactivators was derived from crystal structures of complexes between AChE and various ligands. The molecular docking results are in contrast with non-competitive inhibition implied by reversible inhibition measurements, which usually suggests inhibition of PAS. However, a recent study (27) showed that an overwhelming number of reported mixed/non-competitive inhibitions are artefacts resulting from assumptions inherent to the steady-state model in combination with limitations imposed by most common reversible enzyme inhibition assays. In essence, the underlying mechanism of reversible enzyme inhibition is predominantly competitive, but only biochemical/biophysical methods, which we did not employ in our study, could explain exactly how.

Our choice of the 6,7-dimethoxy-1,2,3,4-tetrahydroisoquinoline group as substituent was governed by the versatility of the THIQ platform which offers a tunable, drug-like framework to support both blood-brain barrier (BBB) permeability and AChE/BChE engagement. Though *in vitro* measurements demonstrated that the introduced substituent did not improve inhibition potency of the lead compound (1), the tested compound (2) retained low micromolar K_I_ for both enzymes and the selectivity for BChE. As the viability of hepatocellular carcinoma (HepG2) and healthy HEK293 cell lines was but slightly affected, and that of human neuroblastoma cells (SH-SY5Y) cells not at all, our findings suggest a promising action of the test compound in the CNS.

## CONCLUSION

Taken together, our evidence supports the need for continued development of uncharged, triple-binding ligands as candidates for selective AChE/BChE reversible inhibition and CNS action. Our study confirms that engaging multiple subsites within the cholinesterase gorge with all three modular elements of the *N*-substituted 2-hydroxyiminoacetamide can lead to its efficient binding. However, the modification of the lead compound (1) from the previous study by introducing 6,7-dimethoxy-1,2,3,4-tetrahydroisoquinoline group as PAS-binding substituent did not further enhance the inhibition potency of the test compound (**2**). Though molecular docking studies predicted that all three modular elements of the test compound **2** would engage in non-bonding interactions with the residues outlining the active site gorge of both AChE and BChE, the predicted binding orientations should ideally be confirmed by X-ray crystallography. The obtained cellular profile of the test compound suggests that triple-binding *N*-substituted 2-hydroxyiminoacetamides can serve as a platform for the development of non-toxic CNS-active ChE inhibitors.
